# The Electroanatomic Volume of the Left Atrium as a Determinant of Recurrences in Patients with Atrial Fibrillation After Pulmonary Vein Isolation: A Prospective Study

**DOI:** 10.3390/biomedicines13010007

**Published:** 2024-12-24

**Authors:** Amaia Martínez León, David Testa Alonso, María Salgado, Ruth Álvarez Velasco, Minel Soroa, Daniel Gracia Iglesias, David Calvo

**Affiliations:** 1 Arrhythmia Unit, Central University Hospital of Asturias, 33011 Oviedo, Spain; amaiamtzleon@gmail.com (A.M.L.); testa84@gmail.com (D.T.A.); msalgadobarq@gmail.com (M.S.); rutalvarez3@gmail.com (R.Á.V.); miguelsoroao@gmail.com (M.S.);; 2 University Hospital of Navarra, 31008 Pamplona, Spain; 3 Institute of Health Research of the Principality of Asturias-Foundation for Health Research and Innovation of the Principality of Asturias (ISPA-FINBA), 33011 Oviedo, Spain; 4 Arrhythmia Unit, San Carlos Clinical Hospital Health Research Institute (IdISSC), 28040 Madrid, Spain; 5 Biomedical Research Center in Network, Cardiovascular Diseases (CIBERCV), 28029 Madrid, Spain

**Keywords:** atrial fibrillation, catheter ablation, echocardiography, electroanatomic mapping

## Abstract

**Background/Objectives**: Catheter ablation for atrial fibrillation (AF) is a well-established therapeutic approach for maintaining sinus rhythm, though its efficacy remains suboptimal in certain patients. The left atrium (LA) volume, commonly assessed through transthoracic echocardiography (TTE), is a recognized predictor of AF recurrence after pulmonary vein isolation (PVI). However, the complex three-dimensional structure of the LA makes precise measurement challenging with traditional TTE techniques. Electroanatomic mapping (EAM) offers a more accurate evaluation of LA geometry and volume, which may enhance the prediction of ablation outcomes. **Methods**: This prospective study included 197 patients with AF who were referred for PVI to our center (Hospital Universitario Central de Asturias, Spain) between 2016 and 2020. All participants underwent pre-ablation TTE and EAM to assess the electric active volume (EAV) of the LA. Clinical follow-up included regular Holter monitoring and electrocardiograms to detect AF recurrences. **Results**: The mean age was 56.3 ± 9.67 years, and 34% had persistent AF. The mean LA volumes measured by TTE and the EAV by EAM were 62.86 ± 15.58 mL and 126.75 ± 43.35 mL, respectively, with a moderate positive correlation (r = 0.49, *p* < 0.001). AF recurrences were observed in 51.27% of patients over a 36 ± 15-month follow-up period. Cox regression analyses (univariate and multivariate), Kaplan–Meier curves and log-rank tests were used to illustrate freedom from atrial arrhythmia during follow-up. Both EAV by EAM and TTE volumes were significant predictors of AF recurrence in the univariate analysis (HR 1.002 [1.001–1.003], *p* = 0.033 and HR 1.001 [1.006–1.012], *p* < 0.01, respectively). Among clinical variables, persistent AF was significantly associated with a higher risk of recurrence (HR 1.17 [1.096–1.268], *p* = 0.02). **Conclusions**: EAV of the LA assessment by EAM demonstrates a significant correlation with TTE measurements and is a predictor of AF post-ablation recurrence. In patients selected for catheter ablation, EAV by EAM provides additional insights that could contribute to therapeutic decision-making and risk stratification of AF recurrences.

## 1. Introduction

Atrial fibrillation (AF) catheter ablation is a well-established interventional technique for cardiac rhythm control strategy, but it presents suboptimal results in specific patient profiles [1]. Given the high recurrence rate in patients with persistent AF, and up to 20% in those with paroxysmal AF, regardless of whether radiofrequency or cryoballoon ablation is used, additional strategies should be investigated to better identify patients and the ablative techniques they would most likely respond to successfully [2].

Several epidemiological studies have identified various risk factors present in most patients who develop AF, such as hypertension, diabetes mellitus, obstructive sleep apnea, heart failure, obesity, aging, or endocrine disorders [3,4,5]. These comorbidities predispose patients to structural and histopathological changes in the left atrium (LA), known as atrial cardiomyopathy, which provides the appropriate substrate for the development of AF [6]. In this regard, the size of the left atrium (LA), an indirect parameter of atrial cardiomyopathy, has been established as one of the main predictors of recurrences after Pulmonary Vein Isolation (PVI) [7].

Transthoracic echocardiography (TTE) is currently the most frequently used method to determine LA size. It has been demonstrated that echocardiographic volume measurement using the biplane disk summation technique more closely approximates the true size of the left atrium than the anteroposterior diameter [8,9,10,11,12]. However, in clinical practice, the most widely used dimension is still the LA anteroposterior diameter in long-axis view [13]. As no electrical information is collected, one limitation of TTE measurements is the lack of information about the extent of the relevant tissue related to the AF substrate, such as the extent of the myocardial sleeves into the pulmonary veins.

On the other hand, electroanatomic mapping (EAM) primarily focuses on the precise visualization of mapping catheters without the use of fluoroscopy, allowing for the creation of 3D models of specific cardiac structures, such as the LA, for the evaluation of its geometry and volume [5]. These models, become more accurate as more data points are collected. The latest versions of EAM systems include tools that facilitate the quick and automated creation of these multi-point models during catheter movement. Multipolar catheters further enhance the process by enabling rapid reconstruction of heart chambers and real-time marking of specific anatomical locations. EAM systems also record electrical information at specific points, which can be used for further mapping analyses, including the color-coded visualization of the referenced electrical activation sequence (“activation mapping”) and the unipolar/bipolar electrograms as part of “fractionation mapping” and “voltage mapping” on the model surface. This technique offers the advantages of reduced fluoroscopy exposure and shorter procedure times [14].

In this respect, EAM could be useful as a more realistic marker of the degree of atrial cardiomyopathy and the expected efficacy of a PVI procedure. Diffuse interstitial fibrosis of the LA, often encounters an enlarged LA, contributes to the dispersion of AF-maintaining mechanisms beyond the antrum of the pulmonary veins (PVs) [15], potentially explaining the failure of PV antrum isolation (PVI) in the context of extensive atrial enlargement and remodeling. For example, EAM enables the identification of low-voltage regions, which are linked to areas of fibrosis, increased intercellular space, myofibril loss, and reduced myocardial nuclear density. Several studies support the prognostic value of assessing the low-voltage atrial area through voltage maps during invasive EAM [16,17].

Here, we postulate that the electrically active volume (EAV) using EAM, newly defined as the LA volume determined by the whole electrically identified tissue, can accurately reconstruct the electrically relevant three-dimensional and highly complex structure of the LA, providing a more realistic overview of the substrate, promoting and perpetuating AF in patients. This approach could be particularly valuable to tailor ablation techniques (i.e., the decision on when to proceed to extensive or extrapulmonary ablation procedures) and risk stratification of recurrences after the procedure.

## 2. Materials and Methods

### 2.1. Study Population

A prospective registry was carried out, including all patients selected for AF ablation at our center, Hospital Universitario Central de Asturias (Spain), in accordance with the recognized recommendations of the European Society of Cardiology for every period of time [7,18].

Inclusion criteria were age, over 18 and under 75 years, and symptomatic paroxysmal or persistent AF despite pharmacological treatment or intolerance to medications. On the other hand, exclusion criteria comprised the presence of significant associated comorbidities such as stage 4 or higher chronic kidney disease, moderate to severe chronic obstructive pulmonary disease, an active malignancy, or a severe active infection.

**Figure 1:**
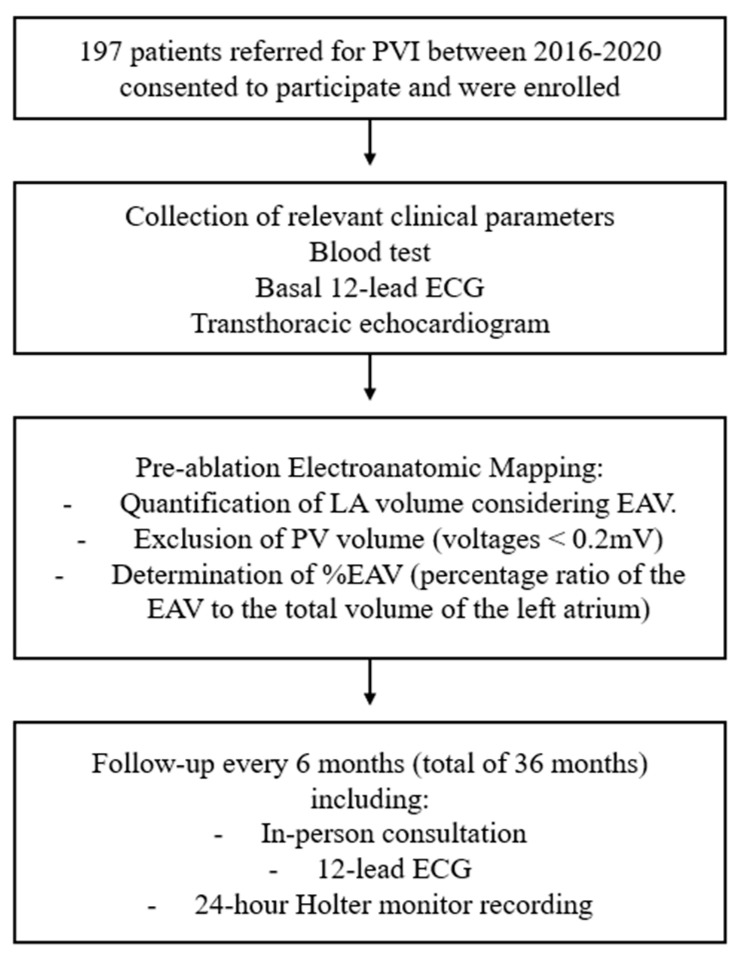
Study design flowchart. EAV, electrically active volume; ECG, electrocardiogram; PV, Pulmonary veins; PVI, Pulmonary vein isolation.

All patients underwent comprehensive blood tests, and the most significant clinical parameters before the ablation procedure had been recorded. Clinical follow-up consisted of in-person consultations every 6 months, including a 24-h Holter monitor recording and a 12-lead electrocardiogram (ECG) prior to each visit. To assess clinical recurrences, arrhythmic episodes occurring within the first 3 months post-ablation were not considered (blanking period). AF recurrence was defined as documentation of AF on a 12-lead ECG or AF episode greater than 30 s on a 24-h Holter monitor (Figure 1).

### 2.2. Electrocardiographic Analysis

A 12-lead resting ECG was recorded for all patients before and after the ablation procedure with the aim of analyzing potential electrocardiographic predictors of arrhythmic recurrence [19]. The analysis was conducted blindly, focusing specifically on the duration of the P wave and the presence of interatrial block criteria [20] (Figure 1).

### 2.3. Echocardiographic Analysis

Before undergoing PVI, all patients had a standardized transthoracic echocardiogram (TTE) according to the guidelines established by the European Society of Cardiac Imaging [13,21]. This included a comprehensive analysis of LA parameters, with measurements taken for its diameter, area, and volume (Figure 1).

### 2.4. Electroanatomic Mapping Procedure

**Figure 2:**
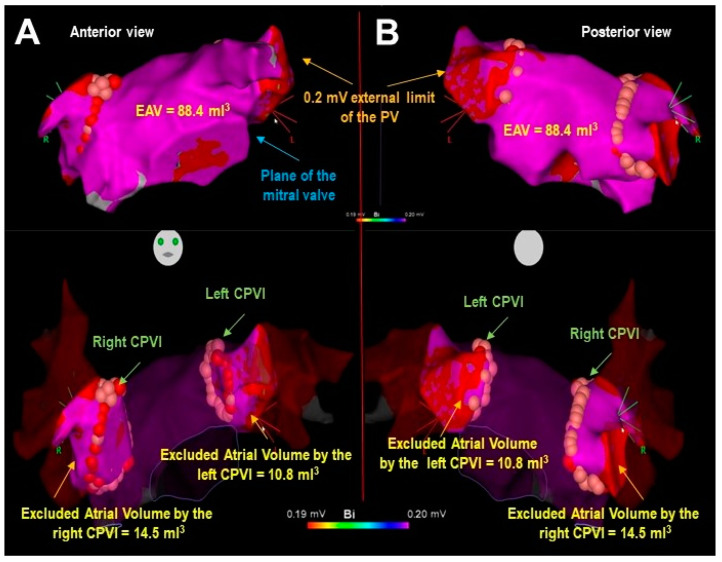
Sample case of left atrial electrically active volume (EAV) quantification using electroanatomic mapping. (**A**) Anterior view. (**B**) Posterior view. Upper panels display EAV quantified between the plane of the mitral valve and the external limit of the pulmonary veins (PV) set by voltage mapping at 0.2 mV. Lower panels display quantification of excluded atrial volumes by the circumferential pulmonary vein isolation (CPVI) lines.

The ablation procedure was performed using the Carto3^®^ EAM system (Biosense Webster, Inc., Irvine, CA, USA, EE.UU.), along with the Biosense SmartTouch^®^ ablation catheter (Biosense Webster, Inc., Irvine, CA, USA, EE.UU.) and the Biosense Pentarray^®^ diagnostic catheter (Biosense Webster, Inc., Irvine, CA, USA, EE.UU.). Following pre-ablation EAM, the LA volume was quantified by considering only the electrically active volume (EAV) and excluding the volume corresponding to the pulmonary veins with voltages below 0.2 mV (Figure 2). After completing the isolation, the excluded atrial volume was also quantified, defined as the EAV determined between the external limit of the pulmonary vein set at 0.2 mV and a plane adjacent to the circumferential ablation line. The percentage ratio of the EAV to the total volume of the left atrium (%EAV) was then determined.

According to protocol and current clinical guidelines at each period [7,18], most of the patients received anticoagulation for 3–4 weeks before the procedure and continued it for 2 months afterward.

### 2.5. Statistical Analysis

Statistical analysis was performed using “R” statistical software [22]. Continuous variables are expressed as mean and standard deviation and categorical variables as absolute value and percentage. For comparison between groups with and without AF recurrence during follow-up, a Student’s t-test was used for continuous variables (after confirming normality) and a Chi-square test for categorical variables: (for absolute frequencies less than five, Fisher’s exact test was used instead).

To evaluate the predictive value of AF recurrence, we applied a multi-step statistical approach. Initially, each variable was analyzed individually using univariate Cox regression to identify those significantly associated with AF recurrence. Variables showing significance in this preliminary screening were subsequently included in a multivariate Cox regression model, which allowed for the assessment of the combined effects of multiple predictors on the time to AF recurrence while adjusting for potential confounders.

Kaplan–Meier survival curves were constructed to illustrate the cumulative freedom from atrial arrhythmia during the follow-up period. The log-rank test was performed to compare recurrence-free survival between groups, identifying any statistically significant differences.

To evaluate the consistency and potential interchangeability of LA volume measurements, Pearson’s correlation coefficient was used to assess the relationship between LA volume as measured by TTE and the EAV by EAM.

Receiver operating characteristic (ROC) curve analysis was conducted to compare the predictive capacity of LA volume for AF recurrence based on measurements from TTE and EAV by EAM. The area under the ROC curve (AUC) was calculated for both methods, providing a quantitative assessment of each parameter’s ability to predict AF recurrence post-ablation and offering insight into the discriminative power of each LA volume measurement. Both AUCs were compared using the DeLong statistical test.

## 3. Results

### 3.1. Baseline Characteristics

A total of 197 patients referred for PVI between 2016 and 2020 were included, of which 74.11% were men. The mean age was 56.3 ± 9.67 years: 33.17% of them had hypertension, and 15.12% were diagnosed with obstructive sleep apnea. Moreover, 22.93% of the patients had previously undergone PV ablation. It is worth noting that most patients had a CHA2DS2-VASc score of 0 (85 patients, 43%) or 1 (70 patients, 35.5%), and this is the reason for the low rate of prior anticoagulation before the procedure. By “cardiomyopathy”, we are referring to all patients who were diagnosed with a cardiac condition beyond AF itself. Among these, the three most common conditions we encountered were heart failure (10.73%), coronary artery disease (2.93%), and valvular heart disease (4.39%). Other cardiomyopathies, such as hypertrophic cardiomyopathy, Brugada syndrome, or aortic dilation, were less frequent in our sample and therefore are not specified separately. The rest of baseline characteristics of the study population are shown in Table 1.

### 3.2. Correlation of LA Volumes

**Figure 3:**
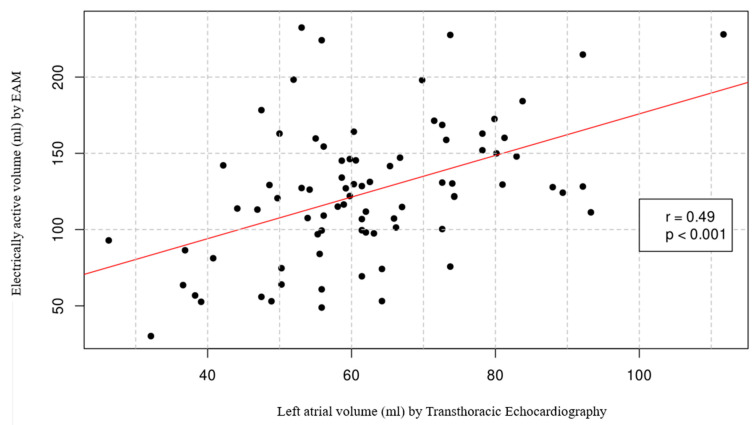
Comparison between Left Atrial volume measured with transthoracic echocardiography and the electrically active volume by EAM. The regression line is plotted in red. EAM: electroanatomic mapping.

The mean LA volume measured by TTE was 62.86 ± 15.58 mL, and the EAV by the EAM was 126.75 ± 43.35 mL, with an excluded volume percentage of 28.26 ± 7.78%. A positive linear relationship (r = 0.49) was observed between volume measurements by TTE and EAV by EAM, which was statistically significant with *p* < 0.001, although of weak magnitude (Figure 3). In 33 patients (15.74%), it was not possible to measure EAV by EAM, while in 99 patients (50.25%), the LA volume measurement by TTE was not reliable.

### 3.3. Predictors of AF Recurrence

Table 2 presents the comparison of analyzed parameters between the group that experienced arrhythmic recurrence and the group that remained event-free during follow-up, as assessed in the univariate Cox regression analysis. Arrhythmic recurrence occurred in 101 patients (51.27%) over a 36 ± 15-month follow-up period. In univariate logistic regression, EAV by EAM (HR 1.002 [1.001–1.003], *p* = 0.033) and LA volume by TTE (HR 1.001 [1.006–1.012], *p* < 0.01) were predictors of AF recurrence. Among the analyzed clinical variables, only the presence of persistent AF predicted a higher risk of recurrence (HR 1.17 [1.096–1.268], *p* = 0.02). The LA anteroposterior diameter by TTE was not a recurrence predictor. In the multivariate Cox regression analysis, only the LA volume measured by TTE remained an independent predictor of AF recurrence (HR 1.023 [1.011–1.035], *p* = 0.05).

**Figure 4:**
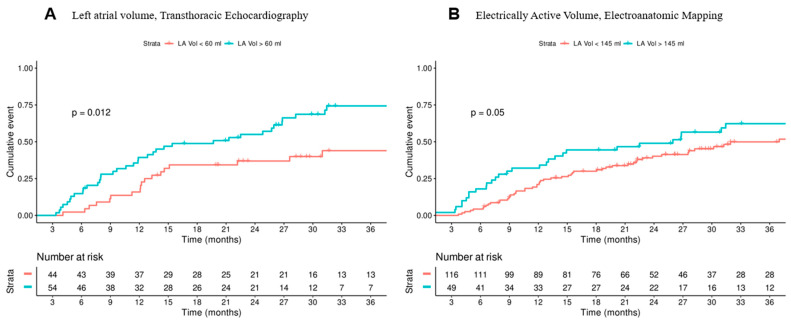
Cumulative risk of Atrial Fibrillation recurrence during follow-up depending on Left Atrial (LA) volume. (**A**) LA volume measured by transthoracic echocardiography; red indicates LA volume <60 mL and blue indicates LA volume >60 mL. (**B**) Electrically active volume (EAV) measured by electroanatomic mapping; red indicates EAV <145 mL, and blue indicates EAV >145 mL.

In the survival analysis, patients with a smaller LA volume showed greater AF-free survival, whether measured by EAV by EAM (23.27 ± 13.59 months if LA < 145 mL vs. 24.11 ± 16.3 months if LA > 145 mL) or by TTE (26.22 ± 18.43 months if LA < 60 mL vs. 27.26 ± 19.09 months for LA > 60 mL (Figure 4).

**Figure 5:**
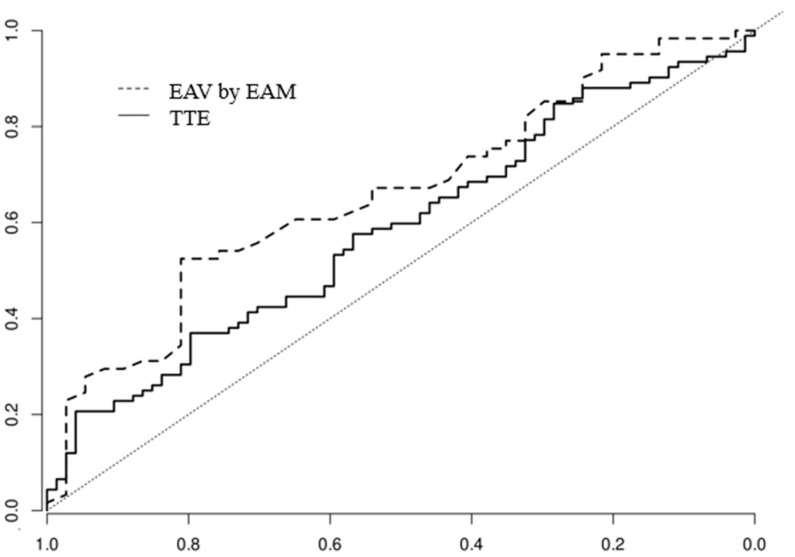
Comparative ROC curves of measurements by transthoracic echocardiography (TTE) and electrically active volume measured by electroanatomic mapping (EAV by EAM), demonstrating similar predictive capabilities for both diagnostic tests.

The ROC curves used to predict AF recurrence after ablation indicate no statistically significant difference between the AUC for EAV by EAM (AUC = 0.65) and the LA volume by TTE (AUC = 0.6), with *p* = 0.49, as shown in Figure 5.

## 4. Discussion

The most interesting results from this study are the correlation between the LA volume measured by TTE and EAV by EAM using the Carto3^®^ EAM system (Biosense Webster, Inc., Irvine, CA, USA, EE.UU.) and the relationship of volumes obtained by EAM with AF recurrences. Given that LA volume measurements by EAM and TTE are shown to be comparable, the multivariate analysis cannot be statistically significant for both measurements. Despite both techniques displaying similar diagnostic yield, the echocardiography volume was not possible to be obtained in a higher part of the patients due to constrains of the technique. Other predictors of AF recurrence analyzed in our sample are consistent with currently available evidence, with the exception of the LA anteroposterior diameter which displays no predictive capabilities.

Catheter ablation of paroxysmal and persistent AF is a well-established treatment for preventing recurrences. It has demonstrated to be both safe and superior to antiarrhythmic drugs in maintaining sinus rhythm (SR) and ameliorating symptoms [23,24,25,26]. However, the rate of arrhythmic recurrence after this procedure is not negligible and depends on a complex interaction of factors including the duration and type of AF and comorbidities such as hypertension, diabetes, or sleep apnea, which are modifiable factors [4,5,7].

Moreover, morphological and functional changes in LA tissue are both a cause and consequence of AF, increasing the risk of perpetuating the arrhythmia and promoting recurrence after catheter ablation [6]. To predict the risk of arrhythmic recurrence associated with this atrial cardiomyopathy, several echocardiographic parameters have been evaluated. The most classic and consistently reliable parameter is the anteroposterior diameter of the LA in the parasternal long-axis view ≥50 mm [11,27], although it is an imprecise measure considering that the LA is a three-dimensional structure that in patients with AF often shows some degree of dilation and remodeling. Thus, it has been proposed that an indexed LA volume ≥ 64 mL/m2 obtained by TTE may be a more reliable and consistent measure to predict arrhythmic recurrence, as well as the global atrial longitudinal strain in SR [8,9,10,11,12]. However, echocardiographic measurements are highly dependent on both the patient’s acoustic window and the operator’s proficiency, as is evident in this study where reliable measurements of the LA volume were unobtainable in half of the patients.

While it is true that 2D echocardiographic measurements of the LA volume are relatively easy to perform, their reliability can be questionable in some cases. To obtain the data for our study, we relied on the reports and images analyzed by the cardiac imaging experts at our center. We identified a significant limitation: Obtaining the atrial measurement in the apical two-chamber view necessary for calculating atrial volume using the biplane disk summation algorithm recommended by the current Chamber Quantification Guidelines [13] was challenging in many patients due to the difficulty in acquiring an acceptable echocardiographic window. Consequently, in many reports, the atrial measurements provided were the anteroposterior diameter in the parasternal long-axis view or the area in the apical four-chamber view, rather than the biplane volume. As previously explained, although these measurements also reflect atrial cardiopathy, recent literature suggests that they are less relevant for predicting AF recurrences compared to atrial volume [13,28], and in our study, they were not statistically significant for predicting AF recurrence in the multivariate analysis. Moreover, we would like to emphasize that the volume obtained by 2D echocardiography assumes a three-dimensional measurement of a complex structure with the inherent errors that this can entail.

Advanced imaging studies using Computed Tomography or Magnetic Resonance Imaging have shown promising results [29,30], offering the advantage of being performed before the ablation procedure. Our study emphasizes the need for realistically adjusted volumetric measurements of the complex three-dimensional structure of the LA, as achieved with EAM, which in our experience, could be obtained quickly, easily, and reliably in nearly all patients during the electrophysiological study.

Several studies have demonstrated the prognostic value of the extent of low-voltage areas during invasive EAM. Low-voltage areas are defined as those <0.5mV in SR or <0.24 mV in AF. An extension >5% in paroxysmal AF or >15% in persistent AF increases the risk of recurrence after catheter ablation [16,17]. However, the value of EAV by EAM as a potential predictor of AF recurrence after catheter ablation and its correlation with LA volume measured by TTE had not been analyzed until now. High density EAM is widely used today during invasive electrophysiological procedures [14,15]. It provides opportunities to study accurate geometrical configuration of the left atrium combined with electrical properties, which altogether approximate the extent of atrial remodeling [3,4,6,31]. On the contrary, geometrical characterization itself, as provided by echocardiography or other imaging modalities, lack opportunities to study functional electrophysiological properties which in turn are essential determinants of arrhythmia recurrences. For example, imaging modalities cannot approximate how much of the PV and PV antrum must be included as part of the atrial anatomy. Our study introduces an effective technique to combine geometry and electrophysiology based on accurate anatomy and detailed electrical extension. As previously postulated, EAV by EAM can accurately reconstruct the electrically relevant three-dimensional and highly complex structure of the LA, providing a more realistic overview of the substrate promoting and perpetuating AF in patients and paving the way for better tailoring of ablative techniques and prediction of arrhythmic recurrences following invasive procedures.

### Limitations

This is a single-center study where patient selection for PV ablation is relatively rigorous in terms of age, echocardiographic measures of the LA, and associated comorbidity. Therefore, the rate of AF recurrence after the procedure might be underestimated compared to other centers with more lenient patient inclusion criteria.

Moreover, echocardiographic measurements were performed by different independent operators as part of routine clinical practice and collected prospectively. Although it faithfully reflects the reality of clinical work, it is not free from operator-dependent variability in measurements. Lastly, the extension of scar tissue in the atria is a relevant parameter that has not been addressed in the present study.

## 5. Conclusions

Measurement of EAV by EAM correlates to volume measurement by TTE. Both measurements have proven to be predictors of AF recurrence after a catheter ablation procedure. However, EAV by EAM can offer a more accurate and realistic measurement of LA volume combined with electrical properties, especially since this is a complex structure that, in patients with AF, often shows some level of remodeling. Additionally, this method is easy to perform in the electrophysiology lab, does not have the limitations of acoustic windows, and is less dependent on the operator’s skill compared to TTE. Nonetheless, given that it is an invasive procedure, its role for risk stratification at the outpatient clinic is clearly limited. We postulate that it would be more appropriately reserved for patients already selected for catheter ablation based on clinical indications, where EAM could provide significant additional insights that contribute to therapeutic decision-making (i.e., the decision on when to proceed to extensive or extrapulmonary ablation procedures) and risk stratification of AF recurrences.

## Figures and Tables

**Table 1:** Basal characteristics of analyzed patients. AF, Atrial Fibrillation; EAM, electroanatomic mapping; EAV, electrically active volume; TAPSE, tricuspid annular plane systolic excursion; TTE, transthoracic echocardiography.

	N (%) /Med (SD)
Male	146 (74.11)
Age (years)	56.3 (9.67)
Body mass index (kg/m^2^)	28.88 (6.81)
Hypertension	68 (33.17)
Diabetes	9 (4.39)
Obstructive Sleep Apnea	31 (15.12)
Stroke	10 (4.88)
Creatinine clearance (mL/min)	101.01 (25.58)
Previous diagnosed cardiomyopathy (total) - Heart Failure - Coronary Artery Disease - Valvular Disease	48 (23.41) 22 (10.73) 6 (2.93) 9 (4.39)
Persistent Atrial Fibrillation	70 (34.15)
Prior AF Ablation	47 (22.93)
Concurrent Typical Atrial Flutter	38 (18.54)
Antiarrhythmic (Prior ablation)	112 (54.63)
Beta-blocker (Prior ablation)	134 (65.37)
Oral anticoagulation > 3–4 weeks prior ablation	135 (65.85)
Antiarrhythmic (After ablation)	159 (77.56)
Beta-blocker (After ablation)	131 (63.9)
Oral anticoagulation (After ablation)	196 (95.61)
Number of failed antiarrhythmic drugs	1.04 (0.82)
Left Atrial diameter parasternal long axis (cm)	3.86 (0.6)
Left Atrial volume by TTE (mL)	62.86 (15.58)
Right Atrial minor axis diameter 4-chamber view (cm)	4.55 (2.76)
Left Ventricle Ejection fraction (%)	60.09 (10.09)
TAPSE (cm)	2.6 (1.97)
EAV by EAM (Carto) (mL)	126.75 (43.35)
Excluded EAV (mL)	49.55 (17.18)
Excluded EAV (Percentage)	28.26 (7.78)
P wave duration (ms)	120.99 (15.11)
Biphasic P Wave (inferior leads)	50 (24.39)
Biphasic P Wave (right precordial leads)	45 (21.95)
Interatrial block	33 (16.1)
PR interval (ms)	171.59 (26.68)

**Table 2:** Comparison of analyzed parameters between patients with or without AF recurrence. AF, Atrial Fibrillation; EAM, electroanatomic mapping; EAV, electrically active volume.

	Recurrence	No Recurrence	*p*
	N (%) /Med (SD)	N (%) /Med (SD)	
Male	84 (71.79)	64 (80)	0.254
Age (years)	57.18 (9.59)	55.02 (9.69)	0.126
Body mass index (kg/m^2^)	29.19 (7.97)	28.43 (4.61)	0.405
Hypertension	41 (35.04)	27 (33.75)	0.972
Diabetes	8 (6.84)	1 (1.25)	0.086
Sleep Apnea	18 (15.38)	13 (16.25)	1
Stroke	7 (5.98)	3 (3.75)	0.743
Creatinine clearance (mL/min)	100.39 (26.88)	101.91 (24.02)	0.681
Heart Failure	17 (14.53)	5 (6.25)	0.114
Coronary Artery Disease	2 (1.71)	4 (5)	0.226
Valvular Disease	8 (6.84)	1 (1.25)	0.086
Persistent Atrial Fibrillation	49 (41.88)	21 (26.25)	0.036
Prior AF Ablation	34 (29.06)	13 (16.25)	1
Concurrent Typical Atrial Flutter	24 (20.51)	14 (17.5)	0.732
CHADSVasc	0.96 (0.95)	0.72 (0.94)	0.092
HASBLEED	0.17 (0.4)	0.07 (0.31)	0.057
Antiarrhythmic (Prior ablation)	64 (54.7)	48 (60)	0.554
Beta-blocker (Prior ablation)	79 (67.52)	55 (68.75)	0.979
Oral anticoagulation (Prior ablation)	87 (74.36)	48 (60)	0.048
Antiarrhythmic (After ablation)	94 (80.34)	65 (81.25)	1
Beta-blocker (After ablation)	76 (64.96)	55 (68.75)	0.689
Oral anticoagulation (After ablation)	117 (100)	79 (98.75)	0.848
Number of failed antiarrhythmic drugs	1.03 (0.85)	1.05 (0.78)	0.893
Left Atrial diameter (cm)	3.91 (0.56)	3.82 (0.62)	0.497
Left Atrial area (cm²)	23.64 (5.59)	20.42 (4.91)	0.004
Left Atrial volume (mL)	66.03 (15.61)	57.01 (13.71)	0.004
Right Atrial diameter (cm)	4.29 (2.29)	4.78 (3.2)	0.503
Right Atrial area (cm²)	18.12 (6.62)	16.92 (5.33)	0.397
Left Ventricle Ejection fraction (%)	59 (9.75)	61.76 (10.37)	0.185
TAPSE (cm)	2.77 (2.47)	2.32 (0.58)	0.24
EAV by EAM (mL)	133.17 (45.81)	118.76 (38.93)	0.03
Excluded EAV by EAM (Percentage)	27.91 (7.91)	28.69 (7.65)	0.533
P wave duration (ms)	122.09 (14.15)	119.82 (16.13)	0.45
Biphasic P Wave (inferior leads)	27 (23.08)	23 (28.75)	<0.01
Biphasic P Wave (right precordial leads)	18 (15.38)	27 (33.75)	<0.01
PR interval (ms)	175.02 (28.85)	167.96 (23.93)	0.179

## Data Availability

Data are available upon request from the corresponding author.
